# Authors’ Reply: Comment on “A New Cuffless Device for Measuring Blood Pressure: A Real-Life Validation Study”

**DOI:** 10.2196/12477

**Published:** 2018-10-22

**Authors:** Sebastian Bredie, Tom van de Belt, Harry van Goor

**Affiliations:** 1 Internal Medicine Radboud University Medical Center Nijmegen Netherlands; 2 REshape Center for Innovation Radboud University Medical Center Nijmegen Netherlands; 3 Surgery Radboud University Medical Center Nijmegen Netherlands

We thank van Helmond et al [1] for their critical appraisal of our recent study [2] on the use of the CheckMe, a cuffless blood pressure measuring device. Patient-friendly and easy-to-use blood pressure monitors will make an important contribution to self management of the cardiovascular risk profile. It is however important that we learn how these new devices perform in terms of reliability, accuracy, and to what extent they are usable in current practice of hypertension management.

For this reason, we carried out a very first real life validity study, in which we have followed the conditions of the European Society of Hypertension International Protocol (ESH-IP) as much as possible. This rightly raises a number of questions, which we have also addressed in the article. First of all, cuffless blood pressure measurement involves a completely different technique, using of the pulse transition time measured with a pulse oximeter and the electrical heart signal, measured with electrodes. By calibrating once with a classic bracelet blood pressure monitor, an estimate of the missing vessel wall compliance is made, which is then used as a constant factor for follow-up measurements with the CheckMe. Compared to classic Korotkoff tones or oscillometric measurement, new variables are introduced with the cuffless technique.

At present, a description in the ESH-IP is lacking of the way in which such calibration measurement, necessary for the cuffless blood pressure measurement technique, is to be performed. Therefore, no formal validation study can be performed and it is also not possible to determine whether the CheckMe formally meets the criteria stated in the ESH-IP, as we stated in the article.

In anticipation of expected developments in technology and protocoling, we performed a first 'real life' comparison, in which we performed the calibration measurement and gold standard blood pressure measurement with a frequently used automatic office blood pressure monitor. Outside the ESH-IP we examined whether the position of the CheckMe with respect to the heart, which changed the pulse transition time from heart to fingertip, had an effect on the results. We believe that pioneering work in the field of new techniques for e.g. blood pressure measurement stimulates further development. We fully agree that a formal assessment of accuracy must be made according to the consensus in international guidelines. However, in the absence of in this case the requirement for performing the calibration measurement, only that part of the protocol that applies can be followed.

We appreciate the suggestions made by Helmond et al to also present data in an alternative manner and seriously consider a follow-up manuscript for adequate presentation and interpretation. We also recognize that the blood pressure measurement is not included in the FDA approval of the CheckMe. The required pulse oximetry and ECG recording are included, but as we estimate, for the above reason an FDA approval for blood pressure has never been issued.

We appreciate that our preliminary scientific data on the cuffless blood pressure measurement in persons with a broad range of blood pressure values is correctly assessed. We hope this will trigger a discussion about the use of this promising technique in patient care and will follow formal validation studies based on an adapted protocol [3].
